# Establishment of a rabbit uterine cancer model using VX2 tumor fragments

**DOI:** 10.1371/journal.pone.0344982

**Published:** 2026-03-25

**Authors:** Sohyeon Jeong, Dahye Lim, Jungwon Uhm, Hyun-Woong Cho, Hyun Koo Kim

**Affiliations:** 1 Department of Obstetrics and Gynecology, Korea University Guro Hospital, College of Medicine, Korea University, Seoul, Republic of Korea; 2 Department of Obstetrics and Gynecology, Asan Medical Center, Seoul, Republic of Korea; 3 Department of Thoracic and Cardiovascular Surgery, Korea University Guro Hospital, Korea University College of Medicine, Seoul, Republic of Korea; 4 Department of Biomedical Sciences, Korea University College of Medicine, Seoul, Republic of Korea; SNUH, KOREA, REPUBLIC OF

## Abstract

**Background:**

Preclinical uterine cancer models using the rabbit VX2 system have been described in previous studies; however, they often involve complex procedures such as cell culture, uterine suturing, or imaging validation. This study aimed to establish a technically simple and reproducible rabbit model of uterine cancer using VX2 tumor fragments.

**Methods:**

We established a rabbit uterine cancer model by injecting minced VX2 tumor tissue into the endometrium of New Zealand White rabbits. We first generated VX2 tumors in donor rabbits via subcutaneous thigh injection and harvested them after three weeks. Recipient rabbits were assigned to two cohorts with scheduled assessments at 14 days or 4 weeks post-implantation. Tumor formation was assessed at each time point by intraoperative inspection and histopathological analysis.

**Results:**

In the initial 14-day cohort (n = 8), all rabbits developed well-defined uterine tumors without perioperative complications. Histological analysis confirmed viable tumor growth. No lymph node metastasis or distant spread was observed at the 14-day endpoint. In a separate, extended 4-week cohort (n = 8), all rabbits also developed uterine tumors. This cohort demonstrated tumor progression, with 75% exhibiting retroperitoneal lymph node metastasis, and 37.5% showing peritoneal metastasis.

**Conclusion:**

This study demonstrates the feasibility and reproducibility of a simplified VX2 uterine cancer model using tumor fragments. Furthermore, the model replicates metastatic progression, including retroperitoneal lymph node and peritoneal metastasis, by 4 weeks. The model may serve as a reliable platform for future preclinical studies involving uterine tumor biology and metastatic progression.

## Introduction

Uterine cancer remains a significant health concern worldwide, with its global incidence continuously rising [1,2]. Despite advancements in diagnostic techniques and therapeutic approaches, challenges persist in understanding tumor progression, metastasis, and the response to treatment. Preclinical animal models serve as critical tools for bridging the gap between basic research and clinical applications and enable the development and evaluation of novel treatment strategies [3–5].

The VX2 carcinoma model has been widely used in oncological research because of its rapid growth, vascularity, and ability to be transplanted into different anatomical locations [6–9]. Originally derived from papillomavirus-induced squamous cell carcinoma in rabbits, the VX2 model has been successfully implemented in studies on liver, lung, kidney, and pancreatic cancers [10–16]. Its application in gynecologic oncology remains limited, with few studies focusing on its potential as a model for uterine cancer [17,18]. Few rabbit models of endometrial cancer using VX2 tumor cells have been previously reported, including models with lymph node metastasis and advanced imaging evaluations [19,20]. These studies often involve complex techniques such as in vitro cell propagation, uterine suturing, or advanced imaging infrastructure. Furthermore, some models reported variable tumor take rates or required multi-step cell processing.

Therefore, the objective of this study was to establish a technically simple and reproducible rabbit model of uterine cancer using in vivo-propagated VX2 tumor fragments without cell culture or complex surgical procedure. The study was designed to i) evaluate the initial consistency of tumor formation, and ii) characterize long-term tumor progression and metastatic potential (4-week endpoint). This study focused on validating whether the simplified technique could reliably replicate key clinical features, such as lymph node and peritoneal metastasis, thereby laying the groundwork for future refinement and application of this model in gynecologic cancer research.

## Methods

### Animal selection and ethical considerations

Twenty-two New Zealand White rabbits (female, 2.5–3 kg) were used in this study. Six rabbits served as tumor donors for the thigh tumor model, and sixteen rabbits served as recipients for the uterine cancer model.

**Fig 1:**
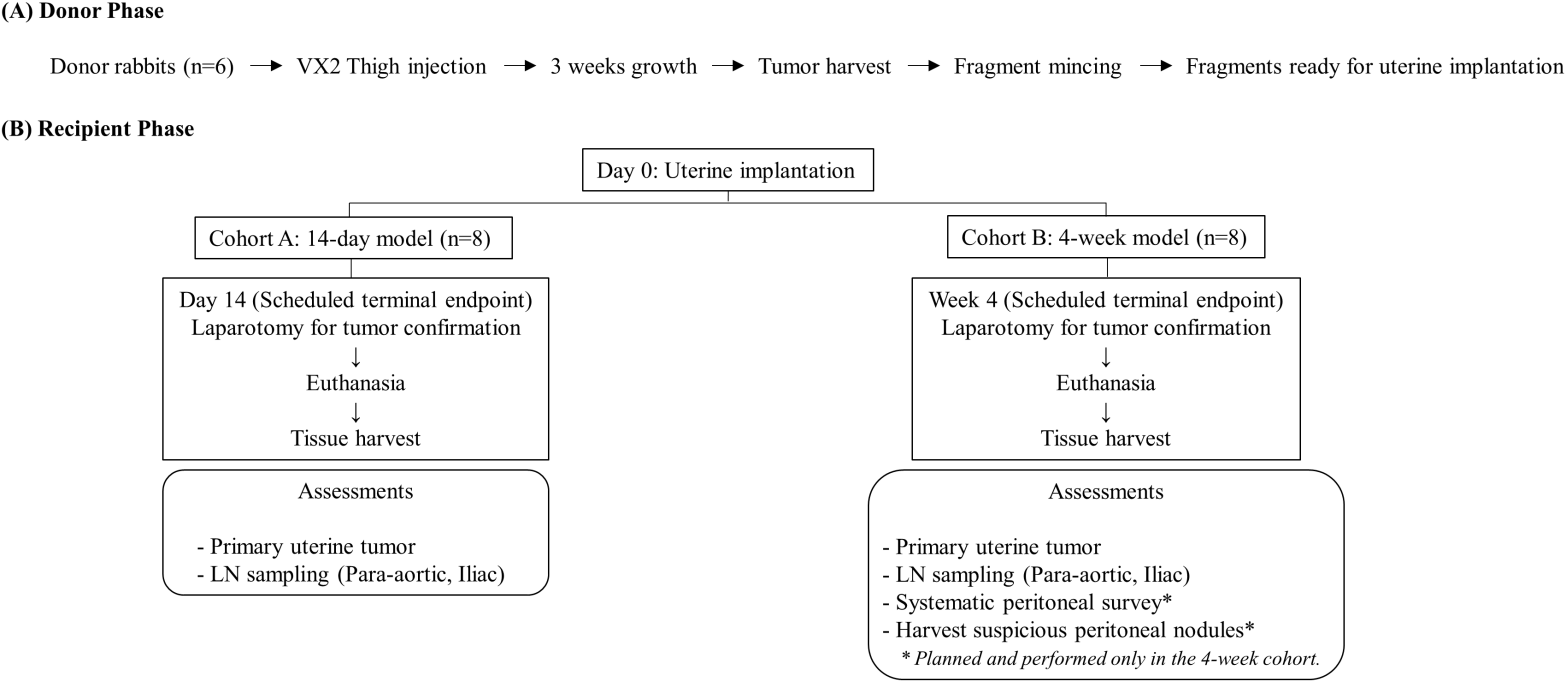
Study design of the VX2 rabbit uterine tumor model. VX2 tumors were generated in donor rabbits (n = 6) by thigh inoculation and harvested after 3 weeks. Tumor tissue was minced into fragments and implanted into the endometrium of recipient rabbits (day 0). Recipient rabbits were assigned to two cohorts: a 14-day cohort (Cohort A, n = 8) and a 4-week cohort (Cohort B, n = 8). At the scheduled terminal endpoint (day 14 or week 4, according to cohort), laparotomy was performed to confirm intrauterine tumor engraftment, followed by euthanasia and tissue harvest. Primary uterine tumors and para-aortic/iliac lymph nodes were collected in both cohorts. A systematic peritoneal survey and collection of suspicious peritoneal nodules were planned and performed in the 4-week cohort.

The 16 recipient rabbits were studied in two cohorts (n = 8 per cohort) to evaluate tumor progression at different time points. The first cohort was euthanized at 14 days post-implantation to assess initial tumor engraftment and feasibility. A second cohort was established to evaluate long-term tumor progression and metastatic potential at 4 weeks post-implantation. The overall experimental workflow and cohort-specific endpoints are summarized in Fig 1.

All experimental procedures were reviewed and approved by the Institutional Animal Care and Use Committee (IACUC) of Korea University Guro Hospital (approval number: KOREA-2021–0213) and conducted in accordance with institutional and national guidelines for the ethical use of laboratory animals. Rabbits were housed individually in temperature- and humidity-controlled environments with ad libitum access to food and water. All animals were monitored at least once daily for general health, appetite, behavior, and signs of infection or distress by trained veterinary personnel.

Humane endpoints were predefined but were not reached before the scheduled endpoints. All rabbits were euthanized at a predetermined time point (14 days or 4 weeks post-implantation, according to their cohort) following intraoperative tumor confirmation. No animals were found dead before the scheduled euthanasia, and no unexpected adverse events occurred. All personnel involved in animal handling, monitoring, and surgical procedures completed institutional training and certification in laboratory animal care and welfare.

### VX2 tumor preparation and implantation

VX2 tumors were first established in the thigh muscles of the six donor rabbits. The initial VX2 tumor fragments were provided by a collaborating laboratory that has previously established this tumor line [21]. These fragments were used to generate thigh tumors in donor rabbits for subsequent implantation procedures described in this study. The donor rabbits underwent a seven-day acclimatization before tumor inoculation. Rabbits were anesthetized with xylazine (5 mg/kg, intramuscular) and Zoletil (15 mg/kg, intravenous, diluted 1:2 in sterile water) to ensure deep anesthesia before tumor implantation. To induce tumor growth approximately 1.0 g of freshly minced VX2 tumor tissue was injected intramuscularly into the thigh muscle of the donor rabbit using a 1 cc syringe fitted with an 18G needle. Tumor development was monitored for three weeks to ensure sufficient tumor mass for subsequent implantation.

**Fig 2:**
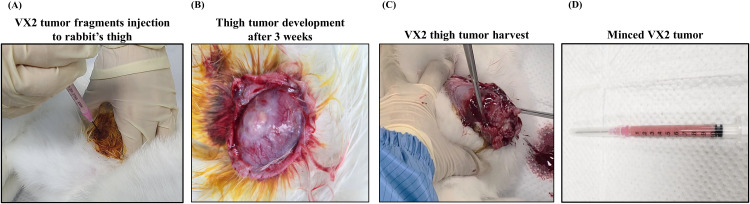
Establishment and processing of VX2 thigh tumor for uterine cancer model implantation. **(A)** Minced VX2 tumor fragments were injected into the thigh muscle of donor rabbits. **(B)** A solid tumor mass developed at the injection site approximately 3 weeks post-inoculation. **(C)** The developed thigh tumor was surgically harvested under sterile conditions. **(D)** The excised tumor was minced into fine fragments suitable for aspiration using a 1 cc syringe with an 18G needle, to be used for implantation into the uterine endometrium of recipient rabbits.

On the day of tumor excision, the donor rabbit was anesthetized using xylazine and Zoletil. The tumors were surgically excised under sterile conditions, yielding approximately 1 g of fresh tissue. The necrotic portions were carefully removed using a sterile scalpel to ensure that viable tumor tissue was utilized for implantation. The peripheral, non-necrotic portion of the tumor was preferentially harvested to maximize viability and consistency across subjects. The excised tumors were immediately minced into small fragments using sterile scissors until the pieces were sufficiently small to be injected through an 18G needle attached to a 1 cc syringe. No enzymatic digestion, filtration, or in vitro cell culture was performed. All tumor fragments were directly derived from in vivo-grown VX2 tumors in donor rabbits and were used immediately after harvest to preserve cellular viability and native tissue architecture. Freshly minced VX2 tumor fragments were implanted immediately after excision without prolonged storage or cooling (Fig 2**).**

Following tumor excision, the donor rabbits were euthanized using carbon dioxide (CO₂) inhalation, followed by confirmation of death to prevent unnecessary suffering and to comply with ethical guidelines for animal research. The carcasses were stored in a designated facility in accordance with institutional policies.

### Surgical procedure for tumor implantation

The recipient rabbits were anesthetized with xylazine and Zoletil. The depth of anesthesia was monitored through reflex testing and by maintaining a stable heart rate. The rabbits were placed in the supine position on a heated surgical table to keep their body temperature maintained during surgery. Midline laparotomy was performed under sterile conditions to expose the uterus. The uterine horn was gently exteriorized, and minced tumor fragments (1 g) were carefully injected into the endometrium of one randomly selected uterine horn (left or right) depending on surgical accessibility using an 18G needle attached to a 1 cc syringe. Care was taken to ensure that the uterus did not perforate during implantation.

**Fig 3:**
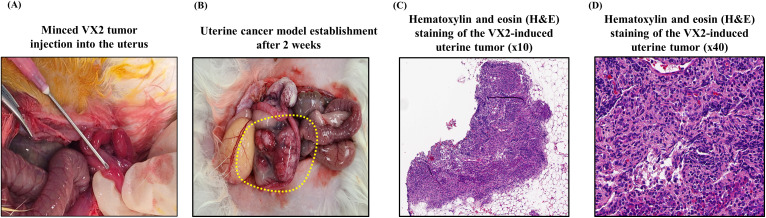
Establishment and histological validation of a rabbit uterine cancer model using VX2 tumor fragments. **(A)** Minced VX2 tumor tissue was injected into the endometrium of the uterine horn using a syringe. (B) 14 days after implantation, macroscopic examination confirmed the formation of localized uterine tumors (yellow dotted line). **(C)** Hematoxylin and eosin (H&E) staining of the excised tumor demonstrated viable VX2 tumor cells and solid tumor architecture within the uterus. (x10) **(D)** Hematoxylin and eosin (H&E) staining of the excised tumor demonstrated viable VX2 tumor cells and solid tumor architecture within the uterus. (x40).

The abdominal wall was closed in layers using 4−0 absorbable sutures for the peritoneum and non-absorbable 3−0 nylon sutures for the skin. No additional hemostatic agents or electrocautery was used; hemostasis was achieved through simple suturing. The rabbits received postoperative analgesia (ketoprofen, 5 mg/kg, subcutaneously) and antibiotics (gentamicin, 2.5 mg/kg intramuscularly) for three days. Animals were housed individually and monitored for pain, infection, or distress. Additional postoperative care included maintaining a stable temperature and regularly monitoring food and water in**take (**Fig 3A**).**

### Tumor confirmation and metastatic assessment via intraoperative exploration

After 14 days or 4 weeks, according to the cohort, intraoperative exploration confirmed tumor formation. The rabbits were re-anesthetized using the same protocol, and a second midline laparotomy was performed to visualize the tumor within the uterus directly. The tumor size and localization were documented before histopathological analysis (**Fig 3B–D).**

**Fig 4:**
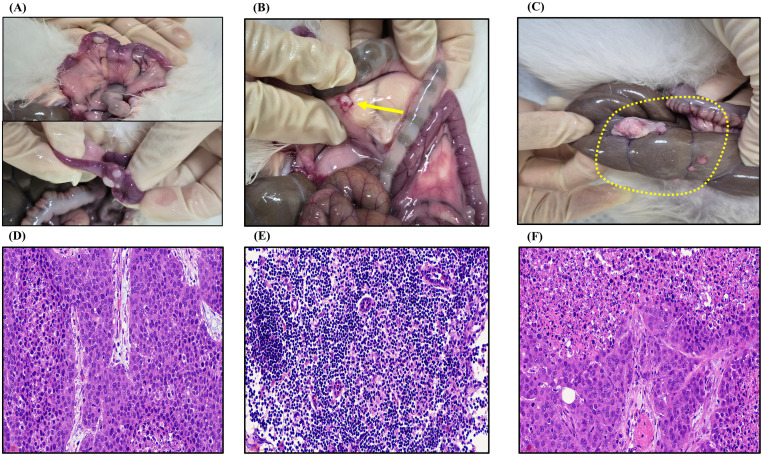
Tumor progression and metastatic validation at 4 weeks. **(A)** Macroscopic view of the primary uterine tumor at 4 weeks. **(B)** Representative image of retroperitoneal lymph node metastasis (arrow). **(C)** Macroscopic evidence of peritoneal metastasis (yellow dotted line). **(D)** H&E staining confirming viable VX2 carcinoma in the 4-week primary tumor (x40). **(E)** H&E staining confirming metastatic VX2 cells within a retroperitoneal lymph node (x40). **(F)** H&E staining confirming metastatic VX2 cells of peritoneal metastasis (x40).

The uterus and surrounding tissues were excised and fixed in 10% neutral buffered formalin for histological analysis. Hematoxylin and eosin (H&E) staining confirmed the presence of tumors, necrotic patterns, and cellular proliferation. To assess the potential metastatic spread, lymph nodes from the para-aortic and iliac regions were collected, with an average of two-to-three nodes per rabbit. Furthermore, in the 4-week cohort, the abdominal cavity was systematically inspected for peritoneal dissemination. Any suspicious nodules in the abdomen were harvested and fixed for histopathological analysis to confirm metastasis (Fig 4).

## Results

### Tumor establishment and growth

The VX2 tumor model was successfully established in both thigh donors and uterine cancer model rabbits. In all six donor rabbits, VX2 tumors developed consistently in the thigh muscle, reaching an average diameter of approximately 3.6 ± 0.2 cm within three weeks of inoculation, confirming the tumorigenic viability of the harvested VX2 tumor tissue. Approximately 1 g of viable tumor was harvested from each donor under sterile conditions and used for uterine implantation in each recipient rabbit. The excised tumors were viable and suitable for subsequent transplantation into a uterine model.

In the 14-day cohort (n = 8), all eight recipient rabbits successfully developed solid uterine tumors following injection of VX2 tumor fragments into the endometrium. At day 14, intraoperative exploration revealed well-defined tumors in all subjects. The average tumor diameter was 1.8 ± 0.4 cm with an approximate thickness of 0.5 cm, corresponding to a mean volume of 1,270 mm³. Gross morphology and tumor location were consistent across animals, indicating high reproducibility of engraftment. The low coefficient of variation (11.1%) in tumor diameters further supports the consistency of tumor growth among recipient rabbits (S1 Table).

Similarly, intraoperative exploration at 4 weeks confirmed consistent engraftment of uterine tumors in all 4-week cohort recipients (n = 8) (Fig 3A, D).

### Surgical and postoperative observations

No intraoperative or perioperative complications were observed in either cohort. All 16 rabbits tolerated the procedures well, with stable recovery and no mortality. Postoperative care, including analgesia and antibiotics, was effective in preventing signs of pain, distress, or infection. Feeding behavior, body weight, and general activity remained stable throughout the respective study periods.

### Histopathological findings

Histologic examination confirmed the presence of viable VX2 tumor cells, displaying dense solid architecture with regions of central necrosis (Fig 2C, D). Mild inflammatory infiltration was observed in adjacent myometrial tissue, but no invasion beyond the uterus was detected. No lymph node metastasis or distant spread was observed at the 14-day endpoint.

In contrast, the 4-week cohort demonstrated significant metastatic progression. (S2 Table). Histological analysis confirmed metastatic VX2 carcinoma in the retroperitoneal lymph nodes of 6 of 8 rabbits (75%) (Fig 3B, E). Furthermore, peritoneal metastasis was confirmed in 3 of 8 rabbits (37.5%). Across these three animals, multiple nodules were identified on the bowel serosa (n = 1), peritoneal wall (n = 1), and mesentery (n = 2), with one animal exhibiting multi-site peritoneal disease (Fig 3C, F).

## Discussion

This study demonstrated that VX2 tumor fragments can be reliably implanted into the uterus of immunocompetent rabbits, leading to consistent tumor formation with reproducible morphology and histopathological features. Additionally, by extending the observation period to 4 weeks, we have shown that this simplified model effectively replicates tumor progression, including retroperitoneal lymph node and peritoneal metastasis. The success of this minimally manipulated, tissue fragment-based approach highlights its potential utility as a preclinical model for uterine cancer research.

Existing rabbit VX2 uterine cancer models often employ cultured cells, multi-step enzymatic processing, or uterine suturing techniques, which may limit reproducibility and accessibility [19,20]. In contrast, our model uses freshly excised, mechanically processed VX2 tumor fragments without the need for in vitro culture or cell counting. The straightforward surgical protocol produced successful engraftment in all 16 recipient rabbits across both cohorts without complications, suggesting a high degree of technical feasibility.

Current preclinical models of uterine cancer predominantly rely on rodent-based xenograft models, in which human endometrial cancer cells are injected into immunodeficient mice. Although these models have provided valuable insights into tumor biology, they have inherent limitations, including differences in immune responses, tumor microenvironments, and anatomical dissimilarities with the human reproductive system. The rabbit VX2 model offers a more physiologically relevant alternative, given the similarities between the rabbit and human uteri [22]. The ability to induce tumor growth in an immunocompetent host allows the study of immune interactions, which is not possible in traditional xenograft models.

A key strength of this study is the 100% tumor engraftment rate observed in all 16 recipient rabbits (14-day and 4-week cohorts), reinforcing the model’s high reliability and reproducibility. Importantly, this study characterizes the metastatic timeline of this simplified model: while the 14-day cohort confirmed rapid engraftment without metastatic spread, the 4-week cohort demonstrated significant metastatic progression, with 75% retroperitoneal lymph node metastasis and 37.5% peritoneal metastasis. Unlike chemical carcinogen-induced models, which often have unpredictable tumor uptake rates and more extended latency periods [23,24], the VX2 model provides a rapid and reproducible alternative for studying uterine cancer progression. Additionally, this study demonstrated that VX2 tumors can be successfully transplanted without requiring enzymatic digestion or the creation of single-cell suspensions, simplifying the implantation process and minimizing variability between experiments. The immediate transplantation of minced VX2 tumor fragments ensures high tumor viability and avoids the potential cell loss associated with prolonged storage or in vitro manipulation.

Despite its advantages, this model has limitations. First, while the follow-up was extended to 4 weeks to capture metastatic events, this endpoint may not reflect the full spectrum of late-stage disease or deep myometrial invasion. Second, while the total recipient sample size was increased to 16 rabbits, future research with larger cohorts will be needed to enhance the statistical robustness for evaluating therapeutic interventions. Finally, incorporating advanced imaging modalities such as MRI, PET-CT, or ultrasound could provide more detailed insights into tumor vascularity and metabolic activity. Thus, the model’s immediate application in advanced imaging or drug delivery research remains to be validated.

Building on previous VX2 uterine cancer models [19,20], our study defines a key distinctive feature: the combination of technical simplicity with metastatic reliability. We believe this approach complements existing models by offering a technically accessible and clinically relevant metastatic platform for both feasibility testing and for future therapeutic studies.

## Conclusion

In summary, this study establishes a simplified and reproducible VX2 uterine cancer model in rabbits using freshly minced tumor fragments. The model not only demonstrates a 100% tumor engraftment rate but also reliably replicates key features of clinical disease progression, including retroperitoneal lymph node and peritoneal metastasis by 4 weeks. The combination of technical simplicity with predictable metastatic outcomes supports its utility as a robust and clinically relevant preclinical model. This platform can serve as a valuable tool for future investigations into the mechanisms of uterine cancer metastasis and the evaluation of novel therapeutic strategies.

## Supporting information

S1 Table Individual tumor diameter measurements in donor and recipient rabbits. (XLSX)

S2 Table Metastatic characteristics of the 4-week recipient cohort (n = 8). (XLSX)
